# Predicting the F(ab)-mediated effect of monoclonal antibodies in vivo by combining cell-level kinetic and pharmacokinetic modelling

**DOI:** 10.1007/s10928-012-9243-7

**Published:** 2012-03-08

**Authors:** Ben-Fillippo Krippendorff, Diego A. Oyarzún, Wilhelm Huisinga

**Affiliations:** 1 Pharmacology & Drug Development Group, Department of Oncology, Cancer Research UK Cambridge Research Institute, Li Ka Shing Centre, University of Cambridge, Cambridge, CB2 0RE UK; 2 Centre for Synthetic Biology and Innovation, Department of Bioengineering, Imperial College London, London, SW7 2AZ UK; 3 Institut für Mathematik, Universität Potsdam, Wissenschaftspark Golm, 14476 Potsdam, Germany

**Keywords:** Cell-level kinetics, Pharmacokinetic models, Therapeutic proteins, EGFR

## Abstract

Cell-level kinetic models for therapeutically relevant processes increasingly benefit the early stages of drug development. Later stages of the drug development processes, however, rely on pharmacokinetic compartment models while cell-level dynamics are typically neglected. We here present a systematic approach to integrate cell-level kinetic models and pharmacokinetic compartment models. Incorporating target dynamics into pharmacokinetic models is especially useful for the development of therapeutic antibodies because their effect and pharmacokinetics are inherently interdependent. The approach is illustrated by analysing the F(ab)-mediated inhibitory effect of therapeutic antibodies targeting the epidermal growth factor receptor. We build a multi-level model for anti-EGFR antibodies by combining a systems biology model with in vitro determined parameters and a pharmacokinetic model based on in vivo pharmacokinetic data. Using this model, we investigated in silico the impact of biochemical properties of anti-EGFR antibodies on their F(ab)-mediated inhibitory effect. The multi-level model suggests that the F(ab)-mediated inhibitory effect saturates with increasing drug-receptor affinity, thereby limiting the impact of increasing antibody affinity on improving the effect. This indicates that observed differences in the therapeutic effects of high affinity antibodies in the market and in clinical development may result mainly from Fc-mediated indirect mechanisms such as antibody-dependent cell cytotoxicity.

## Introduction

Biotechnologically engineered proteins such as monoclonal antibodies (mAbs) have demonstrated their potential in therapies for cancer and other complex diseases [1]. Due to their ability to specifically bind targets, they allow to modulate specific cellular targets and signaling pathways. Various therapeutic proteins on the market use their binding specificity to inhibit cell surface receptors with critical biologic function. At the same time, many targeted receptor systems also constitute a degradation mechanism for such drugs because binding leads to endocytosis and ultimately degradation of the drug. A thorough understanding of the complex interplay between a drug’s pharmacokinetics and its effect is largely missing.

Empirical or semi-mechanistic compartmental models are typically used to analyze preclinical or clinical pharmacokinetic data of protein drugs [2–6]. In these models, the interaction of the drug with its target is represented by an empirical or semi-mechanistic term, accounting for the saturable degradation capacity of the target system. Further, models of target mediated drug disposition (TMDD) have been proposed as a general semi-mechanistic model for drugs that bind with high affinity and to a significant extent to a pharmacologic target such as an enzyme, receptor, or transporter [7–9]. This is accomplished by describing the target as an additional binding compartment.

In systems biology, detailed mechanistic models of targets at the cell level have proven valuable for identifying potent drug targets [10]. Such mathematical models allow identifying and ranking potential targets in cellular networks for achieving specific downstream effects [11, 12]. A recent prominent example is the use of a kinetic model to identify critical components in ErbB signaling pathways [13] and was the basis for the development of a therapeutic antibody that targets the ErbB3 receptor and is currently in Phase II clinical trials [14].

Linking pharmacokinetic and systems biology modelling approaches allows a multi-level description of the system as a whole. These kinds of systems pharmacology models are therefore increasingly advocated by researchers as well as regulators [15]. A combined model for a drugs’ pharmacokinetic and its cellular effect would be especially valuable for therapeutic proteins where drug effect and pharmacokinetics are inherently interdependent. As models of both, whole-body pharmacokinetics and cellular target dynamics, are becoming more abundant, the main bottleneck in developing multi-level systems pharmacology models is in how to interface the cellular and whole body layers levels.

The objective of this article is to develop a systematic approach to integrate the cellular-level into compartment models of drug pharmacokinetics. Due to their important role in the treatment of cancer, we have developed a cell-level pharmacokinetic/pharmacodynamic model for antibodies antagonistically inhibiting the epidermal growth factor receptor (EGFR). The binding of one of its natural ligands to the EGFR results in the activation of signal transduction pathways that mediate a variety of cellular responses [16] which include cell proliferation, differentiation, survival, and angiogenesis [17]. We illustrate our approach by developing a cell-level PK/PD model for the anti-EGFR therapeutic antibody zalutumumab in cynomolgus monkeys. The model integrates a compartment model developed based on in vivo plasma data for zalutumumab [6], and a receptor trafficking model based on in vitro data of the EGFR [18–24].

mAbs comprise a variable target-specific F(ab) region and aconstant Fc region [4]. The target-specific part recognizes the targeted protein, whereas the constant part is involved in different mechanism which determine the pharmacokinetics as well as trigger indirect therapeutic effects such as triggering antibody-dependent cell cytotoxicity. Using our combined model and integrating preclinical pharmacokinetic data we have investigated in silico the impact of biochemical properties of anti-EGFR antibodies on the F(ab)-mediated inhibitory effect. This new kind of model allows to identify in silico opportunities and limitations for the optimization of biophysical properties of future therapeutic antibodies.

## Theoretical

### Compartment model of in vivo therapeutic antibody pharmacokinetics

**Fig. 1:**
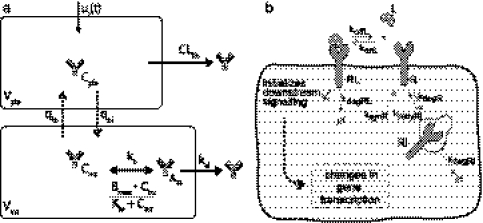
Schematic illustration of the pharmacokinetic model and the kinetic cell-level model.** a** Semi-mechanistic pharmacokinetic compartment model describing the pharmacokinetics of the the mAb zalutumumab in monkeys developed by Lammerts van Bueren et al. [6].** b** Canonical model of ligand-receptor activation and trafficking [19, 29, 20]

The pharmacokinetic part of the multi-level model will be based on Zalutumumab (2F8), an IgG1 antibody against EGFR that inhibits tumor growth in xenograft models and has shown promising results in phase I/II clinical trials [25, 26]. Lammerts van Bueren et al. [6] developed a 3-compartment pharmacokinetic model of zalutumumab in cynomolgus monkeys which accurately describes experimental plasma data for high and low doses (Fig. 1a). In the model, *C*_pla_ and *C*_int_ represent the concentrations of the mAb in plasma (with volume *V*_pla_) and the interstitial space (with volume *V*_int_). *A*_RS_ denotes the amount of drug that is bound to the targeted receptor. The parameters *q*_pi_ and *q*_ip_ denote the transfer flows between the plasma and interstitial compartment, *k*_b_ denotes some large ’artificial’ rate constant that ensures quasi-steady state conditions between the unbound drug concentration in the interstitial space and the drug bound to the receptor. The amount of drug bound to the receptor is modeled in terms of a Michaelis Menten term with *B*_max PK_ denoting the maximal binding capacity of the therapeutic protein to EGFR and *K*_M,PK_ denoting the concentration corresponding to the half-maximal binding capacity. The rate constant of elimination of EGFR by internalization and degradation is denoted by *k*_el_, while the target-independent clearance such as proteolysis in the blood [27] is denoted by CL_lin_. The values of the parameters as used by Lammerts van Bueren et al. are given in Table 1. The rate of change of the molecular concentrations and amount is given by: 1

1 $$ V_{\rm pla}\frac{{\text{d}}C_{\rm pla}}{{\text{d}}t} = - q_{\rm pi} C_{{\rm pla}} + q_{{\rm ip}} C_{{\rm int}}- {\text{CL}}_{{\rm lin}} \cdot C_{{\rm pla}} $$

2 $$\begin{aligned}V_{\rm int}\frac{{\text {d}}C_{\rm int}}{{\text {d}}t}& = + q_{\rm pi}C_{{\rm pla}}- q_{{\rm ip}} C_{{\rm int}}\\ 
&\quad- k_b\left( {\frac{B_{\max,{\rm PK}} \cdot C_{\rm int}}{K_{\rm M,PK} + C_{\rm int}}} - A_{\rm RS}\right) \end{aligned}$$

3 $$ \frac{{\text {d}}A_{\rm RS}}{{\text {d}}t}\,=\,k_b\left( {\frac{B_{\max,{\rm PK}} \cdot C_{\rm int}}{K_{\rm M,PK} + C_{\rm int}}} - A_{\rm RS}\right)-k_{\rm el}A_{\rm RS} $$

**Table 1:** Pharmacokinetic parameters determined in vivo by Lammerts van Bueren et al. [16]

Name	Definition	Value	Unit
*V*_pla_	Plasma volume	70	ml/kg
*V*_int_	Interstitial volume	35	ml/kg
*k*_pi_	Rate constant of plasma-interstitial transport	0.043	1/h
*k*_ip_	Rate constant of interstitial-plasma transport	0.043	1/h
k_b_	Constant thatensures quasi-steady state conditions	0.069	1/h
*B*_max,PK_	Whole-body capacity	2	mg/h/kg
*K*_M,PK_	Half-maximal binding capacity in vivo	$0.5 \cdot 10^{-3}$	mg/ml
k_el_	Elimination of EGFR by internalization and degradation	0.0055	1/h
*q*_pi_	Plasma-interstitial transport	$V_{\rm pla}\cdot k_{\rm pi}$	ml/h
*q*_ip_	Interstitial-plasma transport	$V_{\rm int}\cdot k_{\rm ip}$	ml/h
CL_lin_	Target-independent drug clearance	$V_{\rm pla} \cdot {\rm k_{el}}$	ml/h/kg

Units were converted from mg to nmol using the scaling factor $\text {SF}_{\rm mg\rightarrow\mu mol}=10^6/{\rm MW_{mAbs}}$ with MW_{mAbs = 148000 g/mol, i.e., 1mg = $\text {SF}_{\rm mg\rightarrow\mu mol}\cdot$ nmol

In the above model of Lammerts van Bueren et al., the interaction of zalutumumab with its target (represented by the Michaelis Menten term) accounts for the non-linear feedback of the receptor system on the mAb concentration in the interstitial space, known as receptor mediated endocytosis. With regard to drug effect, the above model does not allow us, however, to analyze the inhibitory effect of zalutumumab on the targeted receptors. Moreover, the Michaelis Menten interaction term is a hybrid parameter in the sense that it combines drug related properties—like binding and dissociation rate constants as well as internalisation rate constants—with receptor system parameters—like receptor synthesis, degradation and internalization [28]. As a consequence, the parameters *B*_max PK_ and *K*_M,PK_ are specific to zalutumuab. An analysis of the impact of changes in the drug-receptor interaction is not feasible with this model, nor is the study of the impact of different cell types, like normal and tumor cells, on the PK and PD of the therapeutic antibody. Both tasks, however, are feasible at the single cell level using kinetic models of the targeted receptor system.

### Kinetic model of in vitro ligand-receptor interaction

To describe the cell-level kinetics we use a canonical model of ligand-receptor activation and trafficking [19, 29, 20] which is parameterized using rate constants that have been experimentally determined and validated in human fibroblast cells [29, 20] (Fig. 1b). The molecular species *R*, *R*_i_, *L* and *RL* denote the numbers of free receptors, free internalized receptors, free extracellular ligand and ligand–receptor complexes per cell, respectively. In the model, the ligand *L* reversibly binds to the free receptors with association rate constant *k*_onL_, and dissociate with rate constant *k*_offL_. The free membrane receptors *R* are internalized with rate constant *k*_degR_ and recycled with rate constant *k*_recyRi_ or degraded with rate constant *k*_degRi_. The ligand–receptor complex is internalized with rate constant *k*_degRL_. The rate of change of the different molecular species is given by:

4 $$ \begin{aligned}\frac{{\text{d}}R}{{\text {d}}t} &= k_{\rm synR}-k_{\rm onL} R \cdot L + k_{\rm offL} \cdot RL - k_{\rm degR} \cdot R \\&\quad+ k_{\rm recyRi} \cdot R_{\rm i} \end{aligned}$$

5 $$ \frac{{\text {d}}R_{\rm i}}{{\text {d}}t} = k_{\rm degR} \cdot R - k_{\rm recyRi} \cdot R_{\rm i} - k_{\rm degRi} \cdot R_{\rm i} $$

6 $$ \frac{{\text {d}}RL}{{\text {d}}t} = k_{\rm onL} R \cdot L - k_{\rm offL} RL - k_{\rm degRL} RL. $$

All molecular species are in number of molecules per cell, except *L* which is in molar concentration. An EGF concentration of $L=2.36\cdot 10^{-3}$ nM was assumed [30].

**Fig. 2:**
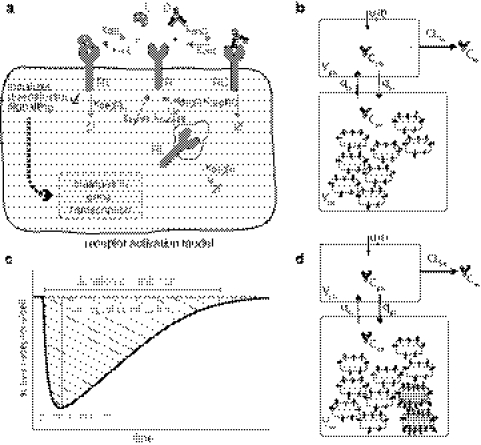
Schematic illustration of the cell-level PK/PD model for analyzing the inhibitory effect on receptor activation of anti-EGFR antibodies.** a** Cell-level receptor model of receptor activation and inhibition. The cellular model describes the transient inhibitory effect of a therapeutic antibody by competitively binding the targeted receptor and thereby decreasing the active ligand-receptor complexes. ** b** Cell-level PK/PD model used to study the trajectory of the drug concentration and the impact of biophysical properties of anti-EGFR antibodies.** c** Three different transient measures of the reduction in the number of active receptors: the integral, the peak, and the duration of inhibition.** d** Extended cell-level PK/PD model including tumor cells with elevated EGFR levels due to alteration of receptor dynamics used to compare the inhibitory effect of therapeutic antibodies on tumor cells and normal cells to optimize tumor specificity

The model of ligand-receptor interaction can easily be extended to account for the drug-receptor interaction by including reactions for drug-receptor association and dissociation (with rate constants *k*_onC_ and *k*_offC_) as well as internalization and subsequent degradation of the drug-receptor complex (with effective rate constant *k*_degRC_), see Fig. 2a. The extended cell-level model including the therapeutic antibody *C*_ex_ [in (nM)] in the extra-cellular space with volume *V*_ex_, and the drug-receptor complex *RC* [in (#molecules)] is given by:

7 $$ V_{\rm ex}\frac{{\text {d}}C_{\rm ex}}{{\text {d}}t} = k_{\rm offC} \cdot \text {SF}_{\rm unit} \cdot RC -k_{\rm onC} \cdot \text {SF}_{\rm unit} \cdot R \cdot C_{\rm ex} $$

8 $$ \begin{aligned}\frac{{\text {d}}R}{{\text {d}}t}&=k_{\rm synR} - k_{\rm onL} R \cdot L - k_{\rm onC} R \cdot C_{\rm ex} + k_{\rm offL} RL \\ &\quad+ k_{\rm offC} RC - k_{\rm degR} \cdot R + k_{\rm recyRi} \cdot R_{\rm i} \end{aligned}$$

9 $$ \frac{{\text {d}}R_{\rm i}}{{\text {d}}t} = k_{\rm degR} \cdot R - k_{\rm recyRi} \cdot R_{\rm i} - k_{\rm degRi} \cdot R_{\rm i} $$

10 $$ \frac{{\text {d}}RL}{{\text {d}}t} = k_{\rm onL} \cdot L \cdot R - k_{\rm offL} RL - k_{\rm degRL} RL $$

11 $$ \frac{{\text {d}}RC}{{\text {d}}t} = k_{\rm onC} \cdot C_{\rm ex} \cdot R - k_{\rm offC} RD - k_{\rm degRC} \cdot RC $$

where the SF_unit_ = 10^9^/*N*_avog_ denotes a scaling factor from [#molecules] to [nmol] with $N_{\rm avo}=6.02 \cdot 10^{23}$ 1/mol denoting Avogardo’s constant. We included those biological processes which are expected to have an impact on the PK of the drug and provide a possibility to link detailed systems biology model of downstream signalling pathway.

To study the inhibitory potential of a therapeutic antibody on a signalling pathway, realistic time-dependent concentration time profiles are essential. As discussed, for many therapeutic antibodies, the targeted system also has an influence on the time-course of the antibody via receptor mediated drug uptake and degradation. Hence, not only has the drug an effect on the receptor system, but also does the receptor system impact on the pharmacokinetics of the drug. As a consequence, we herein propose a novel approach based on integrating the single-cell level into compartment models of antibody PK.

### Linking whole-body and single-cell level

On the whole-body level, the interaction of zalutumumab with its target is represented by a Michaelis Menten term that describes the apparent drug-receptor interactions. At the cellular level, this apparent interaction comprises several kinetic processes, including association and dissociation of the drug-receptor complex, internalization and subsequent degradation of the internalized drug-receptor complex. The assumption underlying our approach is that the apparent drug-receptor interaction on the whole-body level collectively represents the drug-receptor interaction of all relevant cells at the cellular level, i.e., all target–expressing cells that are exposed to the drug. The idea is then to replace the apparent drug-receptor interaction in the compartment model (1–3) by the detailed cell-level model (7–11), scaled from the single-cell to the whole-body level with the number of relevant cells. As a result of this *integration process*, we obtained a cell-level PK/PD model that allowed us to study the pharmacokinetics on the whole-body level and at the same time the inhibitory effect on the cellular level. For the integration, we determined (i) the apparent drug-receptor interaction of a single cell; and (ii) number of all relevant cells *N*_cell_ as the scaling factor that links the apparent drug-receptor interaction of a single-cell to the apparent drug-receptor interaction of the whole-body level.

The *apparent drug-receptor interactions of a single cell* was determined as the reduced description of the cellular model (7–11) using the quasi-steady state assumption on the receptor species *R*, *R*_i_, *RL* and *RC* (see, e.g, [28] for illustrative examples). This resulted in the reduced model for the extra-cellular drug concentration *C*_ex_, the membrane-bound amount of drug *A*_RS_ and the total drug concentration *C*_tot_ = *C*_ex_ + *A*_RS_/*V*_ex_, where *V*_ex_ denotes the extra-cellular volume associated with a single cell:

12 $$ V_{\rm ex}\frac{{\text {d}}C_{\rm tot}}{{\text {d}}t}= - k_{\rm degRC} \cdot {\frac{B_{\rm max,cell} \cdot C_{\rm ex}}{\underbrace{K_{\rm M,cell} + C_{\rm ex}}_{A_{\rm RS}}}} $$

13 $$ C_{\rm ex} = {\frac{1}{2}} \left( C_{D} + \sqrt{(C_{D})^{2}+4K_{\rm M,cell}C_{\rm tot}}\right) $$

with *C*_*D*_ = *C*_tot_ − (*B*_max,cell_/*V*_ex_) − *K*_M,cell_. The parameters *B*_max,cell_ and *K*_M,cell_ denote the maximal binding capacity of a single cell and the concentration of drug at which the binding capacity is half-maximally saturated. The model reduction process also provided us with the relationship between the effective parameters *B*_max,cell_, *K*_M,cell_ and the parameters of the original cellular model (7–11):

14 $$ B_{\rm max,cell} = \text {SF}_{\rm unit} \cdot {\frac{k_{\rm synR}}{k_{\rm degRC}}} $$

15 $$\begin{aligned} K_{\rm M,cell}&=\underbrace{{\frac{k_{\rm degRC}+k_{\rm offC}}{k_{\rm degRC} \cdot k_{\rm onC}}}}_{\rm drug \;specific}  \\&\quad\times\underbrace{\left({\frac{k_{\rm degRi} k_{\rm degR}}{k_{\rm degRi} + k_{\rm recyRi}} + L \cdot {\frac{k_{\rm onL} \cdot k_{\rm degRL}}{k_{\rm offL}+k_{\rm degRL}}}}\right)}_{\rm drug \;independent}\end{aligned}. $$

Note that the maximal binding capacity *B*_max cell_ is only a function of the receptor system and independent of any drug properties, while the Michaelis-Menten constant *K*_M,cell_ depends non-linearly on both, receptor parameters as well as drug parameters. Due to the above relationship (14–15), we are able to explicitly compute the parameters *B*_max cell_ and *K*_M,cell_ based on the in vitro determined parameters *k*_synR_, *k*_degR_, *k*_degRC_, *k*_recyRi_, *k*_onL_, *k*_offL_, *k*_degRL_ of the single-cell model, the in vivo determined EGF concentration *L*, and the drug-specific parameters *k*_onC_, *k*_offC_, *k*_degRC_.

We then determined the *number of relevant cells* *N* as the factor that scales the single-cell binding capacity *B*_max,cell_ to the whole-body binding capacity *B*_max,PK_:

16 $$ \begin{aligned} B_{\rm max,PK} = N_{\rm cell}\cdot B_{\rm max,cell}.\end{aligned} $$

Inserting the relationship (14) of *B*_max,cell_, we obtained

17 $$ N_{\rm cell} = {\frac{k_{\rm degRC} \cdot B_{\rm max,PK}}{k_{\rm synR}\cdot \text {SF}_{\rm unit}}}. $$

Note that all parameter values are known, so we may explicitly determine *N*_cell_ from Eq. 17. In addition, we defined the in vitro-in vivo scaling factor SF_iviv_ between the concentrations of half-maximal binding capacity by

18 $$ \begin{aligned} K_{\rm M,PK} = \text {SF}_{\text{iviv}} \cdot K_{\rm M,cell} \end{aligned} $$

The scaling factor SF_iviv_ accounts for potential differences between conditions in vitro and in vivo.

Next, we present our approach based on a single cell type as a reference cell. We remark that the underlying compartment model including the linear clearance part was taken from the model by Lammerts van Bueren et al. as stated in Eqs. 1–3. In the second part of this article we then extend the cell-level PK/PD model to include multiple reference cell type (tumor and normal cells). Along the same lines, entire distributions of cell types could be integrated e.g., to account for spatial inhomogeneities as they are expected in solid tumors.

### Cell-level pharmacokinetic/pharmacodynamic model

The presented approach allowed a systematic integration of the single-cell level into the compartment model. Based on the number of relevant cells *N*_cell_, we replaced the apparent drug-receptor interaction term in Eqs. 2, 3 by the the drug-ligand-receptor model. The terms accounting for the drug-receptor interaction were scaled to the whole-body level by *N*_cell_. The resulting single-cell PK/PD model is given by:

19 $$ V_{\rm pla}\frac{{\text {d}}C_{{\rm pla}}}{{\text {d}}t} = - q_{{\rm pi}} C_{{\rm pla}} + q_{{\rm ip}}C_{{\rm int}} - {\text{CL}}_{{\rm lin}} \cdot C_{{\rm pla}} $$

20 $$ \begin{aligned}V_{{\rm int}}\frac{{\text{d}}C_{{\rm int}}}{{\text {d}}t}&=  + q_{{\rm pi}} C_{{\rm pla}} - q_{{\rm ip}}C_{{\rm int}} \\&\quad+ N_{{\rm cell}} \cdot \underbrace{\left(k_{{\rm offC}} \cdot {\text {SF}}_{{\rm unit}} \cdot RC - k_{{\rm onC}} \cdot {\text{SF}}_{{\rm unit}} \cdot R \cdot C_{{\rm int}}\right)}_{\rm{whole-body\;single-cell\;level\;interaction}}\end{aligned}$$

21 $$ \begin{aligned} \frac{{\text {d}}R}{{\text {d}}t}&= k_{{\rm synR}} - k_{{\rm onL}} R \cdot L -k_{{\rm onC}} R \cdot C_{{\rm int}} + k_{{\rm offL}} RL  \\ &\quad+ k_{{\rm offC}} RC - k_{{\rm degR}} \cdot R +k_{{\rm recyRi}} \cdot R_{{\rm i}}\end{aligned}$$

22 $$ \frac{{\text {d}}R_{{\rm i}}}{{\text{d}}t} = k_{{\rm degR}} \cdot R - k_{{\rm recyRi}} \cdot R_{{\rm i}} - k_{{\rm degRi}} \cdot R_{{\rm i}} $$

23 $$ \frac{{\text {d}}RL}{{\text {d}}t} = k_{{\rm onL}} \cdot L \cdot R - k_{{\rm offL}} RL - k_{{\rm degRL}} RL $$

24 $$ \frac{{\text{d}}RC}{{\text {d}}t} = k_{{\rm onC}} \cdot C_{{\rm int}} \cdot R - k_{{\rm offC}} RC - k_{{\rm degRC}} \cdot RC $$

that describe the rate of change of the therapeutic antibody in plasma *C*_pla_ and in the interstitial space *C*_int_, the free receptor *R*, the internalized receptor *R*_i_, the drug-receptor complex *RC*, the EGF ligand in the interstitial space *L* and the ligand-receptor complex *RL*. Rather than just re-estimating parameters of the single-cell PK/PD model, the above approach established a mechanistic link between the kinetic model of the receptor system at the single-cell level and the apparent term in the whole-body compartment model. As part of our approach, we provided a systematic way of determining an apparent drug-receptor model from a detailed cell-level description. This has been further elaborated in [28], where we have also shown that the reduced model (12–13) is a more appropriate description of the apparent drug-receptor interaction in the compartment model (1–3), since it eliminates the use of the *artificial* rate constant *k*_b_.

### Measures of receptor saturation, residual activity and inhibition

Receptor saturation by the drug, defined as

25 $$ {\rm receptor\;saturation} = {\frac{RC}{R+RL+RC}}, $$

is often taken as a measure of the inhibitory potential of a drug. We compared receptor saturation with the residual receptor activation

26 $$ {\rm residual\; receptor\;activity} = {\frac{RL}{RL^*}}, $$

defined relative to the pre-treatment level *RL* ^*^ of activated receptors.

We analyzed the impact of mAb treatment of target cells with respect to three quantitative measures. The measures of transient response are illustrated in Fig. 2c and are defined as follows:

- **The integral of inhibition:** Cumulative EGF receptors that are not activated as a consequence of drug treatment. More formally, the integral of inhibition is defined as area under the curve of the active receptors with respect to their steady state pre-treatment level *RL* ^*^, i.e.,
  27 $$ \begin{aligned}  E = \int\limits_{0}^{\infty} (RL^{*} - RL(t)) dt. \end{aligned} $$
- **The peak inhibition:** Maximal reduction in activated EGFR as a fraction of pre-treatment level *RL* ^*^:
  28 $$ {\rm peak} = {\frac{RL^{*} - \min\{ RL\}}{RL^{*}}}. $$
- **The duration of inhibition:** Time needed to recover to 75% of the pre-drug level of activated receptors.

The chosen measures of inhibition resemble important characteristics of drug effect. For small molecule drugs, the integral of inhibition (exposure) is often related to the drug effect, while the peak inhibition or the duration of inhibition (measuring some threshold characteristics) are often related to the side effects.

For different cell types, e.g., normal and tumor cells, we defined the antibody specificity *S* as the ratio of the inhibitory effect on tumor to normal cells. For the three measures of transient response, this amounted to

29 $$ S_E = {\frac{E_{\rm tumor}}{E_{\rm normal}}}, S_p = {\frac{{\rm peak} _{\rm tumor}}{{\rm peak}_{\rm normal}}}, S_d = {\frac{{\rm dur} _{\rm tumor}}{{\rm dur}_{\rm normal}}}, $$

where ’dur’ denotes duration.

### Cell-level pharmacokinetic/pharmacodynamic model with normal and tumor cells

To illustrate our approach and its potential application to different cell types, we integrate tumor cells into the cell-level PK/PD model. For this purpose, we consider only tumor cells that are exposed to the same drug concentration time profile as normal cells. This assumption is expected to hold for tumor cells close to the vasculature, but it is most likely inadequate for cells in solid tumors (in which case model the should be extended to account for a tumor distribution model). To compare the response of normal and tumor cells to anti-EGFR antibodies, we extended our model by integrating a kinetic cellular model representing tumor cells with elevated EGFR levels (Fig. 2d). The rate of change of all molecular species is given as follows, where the subscripts N and T refer to normal and tumor cells:

30 $$ V_{{\rm pla}}\frac{{\text{d}}C_{{\rm pla}}}{{\text{d}}t} = - q_{{\rm pi}} C_{{\rm pla}} + q_{{\rm ip}} C_{{\rm int}} - CL_{{\rm lin}} \cdot C_{{\rm pla}} $$

31 $$ \begin{aligned} V_{{\rm int}}\frac{{\text{d}}C_{{\rm int}}}{{\text{d}}t} &= + q_{{\rm pi}} C_{{\rm pla}}- q_{{\rm ip}} C_{{\rm int}}\\ &\quad+N_{{\rm N}} \cdot \underbrace{\left(k_{{\rm offC}} \cdot {\text{SF}}_{{\rm unit}} \cdot RC_{{\rm N}} - k_{{\rm onC}} \cdot {\text{SF}}_{{\rm unit}} \cdot R_{{\rm N}} \cdot C_{{\rm int}}\right)}_{\rm{normal\;cells}} \end{aligned} $$

32 $$ \begin{aligned}\qquad\quad + N_{{\rm T}} \cdot \underbrace{\left(k_{{\rm offC}} \cdot {\text {SF}}_{{\rm unit}} \cdot RC_{{\rm T}} - k_{{\rm onC}} \cdot {\text{SF}}_{{\rm unit}} \cdot R_{{\rm T}} \cdot C_{{\rm int}}\right)}_{\rm{tumor\;cells}}\end{aligned} $$

33 $$ \begin{aligned} \frac{{\text {d}}R_{{\rm N}}}{{\text {d}}t} &= k_{{\rm synR,N}} - k_{{\rm onL}} R_{{\rm N}} L - k_{{\rm onC}} R_{{\rm N}} C_{{\rm int}}\\ &\quad+ k_{{\rm offL}} RL_{{\rm N}} + k_{{\rm offC}} RC_{{\rm N}} - k_{{\rm degR,N}} R_{{\rm N}}\\ &\quad+ k_{{\rm recyRi}}R_{{\rm iN}} \end{aligned} $$

34 $$ \begin{aligned} \frac{{\text{d}}R_{{\rm i,N}}}{{\text{d}}t}&= k_{{\rm degR,N}} \cdot R_{{\rm N}} - k_{{\rm recyRi}} \cdot R_{{\rm i,N}}\\ &\quad- k_{{\rm degRi}} \cdot R_{{\rm i,N}} \end{aligned} $$

35 $$ \frac{{\text{d}}R_{{\rm LN}}}{{\text{d}}t} = k_{{\rm onL}} L\cdot R_{{\rm N}} - k_{{\rm offL}} RL_{{\rm N}} - k_{{\rm degRL,N}} RL_{{\rm N}} $$

36 $$ \begin{aligned}\frac{{\text {d}}RC_{{\rm N}}}{\text {d}t}& = k_{{\rm onC}} C_{{\rm int}} \cdot R_{{\rm N}} - k_{{\rm offC}} RC_{{\rm N}}\\&\quad - k_{{\rm degRC}} \cdot RC_{{\rm N}}, \end{aligned}$$

37 $$ \begin{aligned} \frac{{\text{d}}R_{{\rm T}}}{{\text{d}}t} &= k_{{\rm synR,T}} - k_{{\rm onL}} R_{{\rm T}} L - k_{{\rm onC}} R_{{\rm T}} C_{{\rm int}}\\ &\quad + k_{{\rm offL}} RL_{{\rm T}} + k_{{\rm offC}} RC_{{\rm T}} - k_{{\rm degR,T}} R_{{\rm T}}\\ &\quad +k_{{\rm recyRi}} R_{{\rm i,T}} \end{aligned} $$

38 $$ \begin{aligned}\frac{{\text {d}}R_{{\rm i,T}}}{{\text {d}}t} &= k_{{\rm degR,T}} \cdot R_{{\rm T}} - k_{{\rm recyRi}} \cdot R_{{\rm i,T}}\\ &\quad - k_{{\rm degRi}} \cdot R_{{\rm i,T}}\end{aligned} $$

39 $$ \frac{{\text {d}}RL_{{\rm T}}}{{\text{d}}t} = k_{{\rm onL}} L \cdot R_{{\rm T}}- k_{{\rm offL}} RL_{{\rm T}} - k_{{\rm degRL,T}} RL_{{\rm T}} $$

40 $$ \begin{aligned}\frac{{\text {d}}RC_{{\rm T}}}{{\text {d}}t}& = k_{{\rm onC}}C_{{\rm int}}\cdot R_{{\rm T}} - k_{{\rm offC}} RC_{{\rm T}} \\ &\quad- k_{{\rm degRC}} \cdot RC_{{\rm T}}.\end{aligned}$$

The parameters for tumor cells are identical to those of normal cells, except for those specified below. Elevated EGFR levels may be caused by a variety of alterations at the target cell level. In the sequel, we analyzed the dynamics response of two tumor cell types that have comparable elevated EGFR levels prior to drug treatment: (i) cells with increased receptor synthesis rate (*k*_synR,N_ vs. *k*_synR,T_); and (ii) cells with decreased receptor internalization (*k*_degR,N_, *k*_degRL,N_ vs. *k*_degR,T_, *k*_degRL,T_). Both tumor cell types have been observed experimentally [31– 34]. We set the number of tumor cells to 1% of the normal cells so that it had little impact on the pharmacokinetics (comparable to the situation in Bleeker et al. [25] in mice). The tumor cell model represents those tumor cells exposed to drug concentrations equivalent to the exposure of cells with normal EGFR levels.

## Methods

For the single-cell PK/PD model with normal cells only, the system is assumed to be in steady state prior to any drug administration, resulting in a number of free receptors *R* ^*^, active receptors *RL* ^*^, and zero drug–receptor complexes *RC* ^*^ = 0. Similarly, for the model with normal and tumor cells, the steady state levels are defined by *R*_N_^*^, *RL*_N_^*^, and *RC*_N_^*^ = 0, *R*_T_^*^, *RL*_T_^*^, and *RC*_T_^*^ = 0.

The response to a bolus dose *C*_0_ is obtained by numerical integration of the corresponding system of ODEs with the following initial conditions

$$ \begin{array}{lll} R_{{\rm N}}(0)=R_{{\rm N}}^{*} & R_{{\rm T}}(0)=R_{{\rm T}}^{*} & C_{{\rm pla}}= C_0\\ RL_{{\rm N}}(0)=RL_{{\rm N}}^{*} & RL_{{\rm T}}(0)=RL_{{\rm T}}^{*} & C_{{\rm int}}=0\\ RC_{{\rm N}}(0)=0 & RC_{{\rm T}}(0)=0. \end{array} $$

For numerical simulations, we used the parameter values given in Table 2. The model was build and simulated using MATLAB (R2011b).

**Table 2:** Parameter values for the EGF receptor system

Name	Definition	Value	Unit	References
k_onL_	Ligand–receptor binding	$7.2\cdot10^{-2}$	1/(nM$\cdot$min)	[20]
k_offL_	Ligand–receptor unbinding	0.34	1/min	[20]
k_degR_	Free receptor internalization	0.03	1/min	[20]
k_degRL_	Ligand–receptor complex internalization	0.03	1/min	[20]
k_R,N_	Receptor expression rate in normal cells	130	Receptors/min per cell	[20]
k_recyRi_	Free receptor recycling	$5.8\cdot 10^{-2}$	1/min	[20]
k_degRi_	Free receptor degradation	$2.2\cdot 10^{-3}$	1/min	[20]
k_onC_	Drug–receptor binding	*k*_onL_	1/(nM$\cdot$min)	
k_degRC_	Drug–receptor complex internalization	0.005	1/h	[6]
MW_mAbs_	Molecular weight	148000	Dalton (g/mol)	

For monkeys, *k*_recyRi_ and *k*_degR_ were multiplied by a factor of 4 and 1/4, respectively to account for species differences

## Results

### Predicting the inhibitory effect of the anti-EGFR therapeutic antibody zalutumumab in cynomolgus monkeys

We determined a single-cell PK/PD model for the anti-EGFR therapeutic antibody zalutumumab in cynomolgus monkeys. The model based on in vivo data for zalutumumab in cynomolgus monkeys [6], in vitro data of human fibroblast cells [29, 20] and determined drug-receptor affinities [35]. Importantly, our approach does not involve any fitting of parameters; all parameter values were either inherited from the original compartment model, determined in vitro, or explicitly calculated.

### Evaluation against in vivo data

**Fig. 3:**
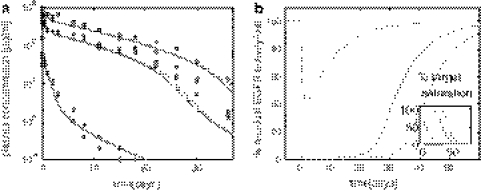
Pharmacokinetics of zalutumumab in cynomolgus monkeys and prediction of the inhibitory effect on a cellular level using themodel depicted in Fig. 2b.** a** Predicted plasma concentration of zalutumumab by the cell-level PK/PD model (*solid line*) and the compartment PK model of Lammerts van Bueren et al. [6] (*dashed line*) for a high, medium and low dose of 40, 20 and 2 mg/kg. The experimental data for zalutumumab in cynomolgus monkeys are marked with circles (40 mg/kg), squares (20 mg/kg) and diamonds (2 mg/kg). Experimental data courtesy of Wim Bleeker, Genmab, Utrecht, The Netherlands.** b** Predictions of the residual EGFR activation per cell based on the cell-level pharmacokinetic model (Fig. 2b) for the high dose (*dashed dotted line*), the medium dose (*solid line*) and the low dose (*dashed line*). The inset depicts the corresponding receptor saturation according to Eq. 25

To evaluate the single cell PK/PD model, we compared our model predictions with the experimental data of zalutumumab in cynomolgus monkeys. Based on the described integration process, we determined the number of relevant cells as $N_{\rm cell}=5.2\cdot 10^9$ and the in vitro-in vivo scaling factor as SF_iviv_ = 2.1. The small scaling factor SF_iviv_ was considered as supporting evidence for the chosen single-cell model. Furthermore, the predicted time-courses of the drug concentrations showed very good agreement for the high, medium and low dose of 40 mg/kg, 20 mg/kg and 2 mg/kg dose (Fig. 3a).

At the same time, the cell-level pharmacokinetic model was used to predict the dynamics of the receptor system upon drug administration (Fig. 3b). In agreement with experimental findings reported in [6] (Table 3), the model predicted that a saturation in monkey tissue which expresses normal receptor levels was established at doses between 2 and 20 mg/kg (Fig. 3b, inset). We considered the agreement between our model and the data available in [6] as validation to proceed confidently in our study. The available data considers 2 and 20 mg/kg doses, and therefore in the sequel we will restrict our analysis to these doses only.

**Table 3:** Pharmacodynamics of zalutumumab in cynomolgus monkeys for different doses as reported in Lammerts van Bueren et al. [6] and predicted by the single-cell PK/PD model (see Fig. 3)

Dose (mg)	In vivo experiment	In silico prediction
2	Not fully saturated	Max. 60 % saturated
20	Fully saturated	100% saturated
40	Fully saturated	100% saturated

### Predicting residual EGFR activity per cell

The cell-level PK/PD model then was used to predict the number of activated receptors over the duration of the treatment, which is difficult to examine in vivo. Our model predicted that the low dose (2 mg/kg) of antibody reduces the number of active receptors by about 35%. The steep initial decrease in receptor activation is followed by a recovery period secondary to a slow reduction of drug concentration (Fig. 3b). On the other hand, the higher dose (20 mg/kg) almost completely inhibited receptor activation for a period of about 20 days. The start of the recovery period coincided with the transition from saturated to linear pharmacokinetics between days 20 and 25. The model therefore suggests that changes in pharmacokinetics mays act as a biomarker for changes in the inhibitory response.

Comparing receptor saturation (25) with residual receptor activity (26), we found that both characteristics only corresponded initially, while at later points in time the receptor saturation underestimated the inhibitory effect of the antibody (e.g. compare with the 20 mg/kg dose after 50 days). This highlights the importance of adopting an integrated kinetic model to translate the binding of the drug into its actual inhibitory effect on receptor activation.

### Impact of drug characteristics on receptor inhibition

One advantage of the cell-level PK/PD model is its ability to predict the impact of drug properties such as the dose, drug-receptor affinity, and drug induced receptor internalization on the inhibitory response under in vivo conditions. We assumed that the target independent PK distribution parameters *V*_pla_, *V*_int_, *q*_pi_ and *q*_ip_ do not change when changing properties of the F(ab) region. Since all the analyzed antibodies are either of IgG1 or IgG2 isotype, their target-independent clearance was also assumed to be identical [36].

#### Affinity and dose

We studied the inhibitory effect for a range of affinities, including those of anti-EGFR mAbs on the market or in clinical development: zalutumumab, panitumumab, cetuximab, IMC-11F8, and nimotuzumab (see Table 4). All these antibodies act antagonistically [37]. Different affinities *K*_D_ were realized by changing the dissociation rate constant *k*_offC_, while the association rate constant *k*_onC_ was assumed to be diffusion-limited and therefore left unchanged. Our analysis focused on the F(ab)-mediated direct inhibitory effect, i.e., on the reduction in the number of activated receptors at the cell membrane.

**Table 4:** Affinities and isotypes of the different therapeutic antibodies against the EGFR. Values taken from Peipp et al. [35]

Antibody	Affinity/avidity (M)	Isotype
Panitumumab	$5\cdot 10^{-11}$	IgG2
Cetuximab	$4\cdot 10^{-10}$	IgG1
IMC-11F8	$3\cdot 10^{-10}$	IgG1
Nimotuzumab	$1\cdot 10^{-9}$	IgG1
Zalutumumab	$7\cdot 10^{-9}$	IgG1

The *K*_D_ (affinity) values were subsequently scaled with SF_iviv_, i.e., $K_{\rm D}(\rm{in vivo}) = \text {SF}_{iviv}\cdot K_{\rm D}(\rm{in vitro})$ to account for differences between conditions in vitro and in vivo. While potentially the scaling factors for *K*_*M*_ (see Eq. 18) and *K*_D_ could be different, we used the same scaling factor SF_*i*_viv due to lack of further information and based on the principle of parsimony. See also footnote to Table 1 of [35], where affinity values are reported

**Fig. 4:**
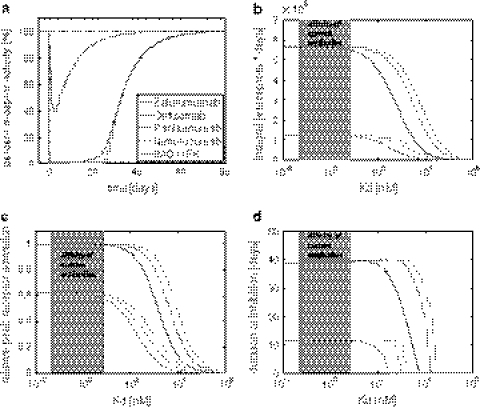
F(ab)-mediated inhibitory effect of different antibodies using the cell-level PK/PD model shown in Fig. 2b.** a** Predicted transient inhibitory effects of five anti-EGFR antibodies on the market or in clinical development with different affinities (see Table 3) for a 20 mg/kg dose (*solid line*) and a 2 mg/kg dose (*dashed line*). The different mAbs show a similar transient inhibitory effect despite their affinities vary 20-fold.** b**–**d** Inhibitory effect resulting from different affinities (KD = 1/affinity = koffC/konC) and downregulation rates (kdegRC). The F(ab)-mediated effect is quantified by three different measures:** b** the integral of inhibition,** c** the peak inhibition, and** d** the duration of inhibition, for the 20 mg/kg dose (*solid line*) and 2 mg/kg dose (*dashed line*). The shaded area indicates the affinity range of the five considered antibodies

The percentage of active receptors over time is shown in Fig. 4a. Despite 20-fold differences in target affinities (see Table 4), the transient inhibition pattern were surprisingly similar. As can be seen in Fig. 4b–d, this phenomenon is a consequence of an effect plateau in the inhibitory responses. For high affinity drugs located in the plateau range, an increased affinity does not translate into a noticeable stronger inhibition. Mathematical analysis of the model (Appendix) for the integral effect suggests that this is a structural feature of the system that does not depend on specific parameter values. Shankaran et al. [19] identified the “consumption parameter”, i.e, the ratio of the dissociation and downregulation rate constants (*k*_degRC_/*k*_offC_), as a key parameter to characterize cell surface receptor systems. It quantifies the likelihood that a drug, upon binding the receptor, is internalized rather than dissociated. We found that this is also an important parameter for antagonistic mAbs, since those with a high consumption parameter are located on the effect plateau such that their F(ab)-mediated direct inhibitory effect could not be further increased. When decreasing the affinity of the mAb, the clearance effect of binding and internalization became less important than the target independent clearance. As a consequence we found that the drug effect, which is related to receptor binding, decreases for lower affinity.

#### Downregulation

Receptor downregulation denotes the drug-induced process of the reducing the number of free receptor at the membrane that is available for binding to the natural ligand. Enforcing receptor downregulation by therapeutic antibodies is argued to be an important part of the drug effect [17]. In Fig. 4b–d, we predicted the inhibitory effect of antibodies with a 5-fold and 10-fold increased internalization rate constant (relative to the rate constant of zalutumumab) for different affinities and low and high doses. We found that for high-affinity antibodies, receptor downregulation only contributes to a negligible extent to the F(ab)-mediated direct inhibitory effect. For medium affinity antibodies, however, an increased downregulation rate constant could increase the direct inhibitory effect to some extent.

#### Tumor cell specificity

Upregulation of EGFR expression and aberrant activation of EGFR has been shown in many human epithelial cancers, including those of the colon, lung, kidney, head and neck, breast, prostate, brain and ovary [38–43]. The extent of overexpression also correlates with a poorer clinical outcome [44, 45].

**Fig. 5:**
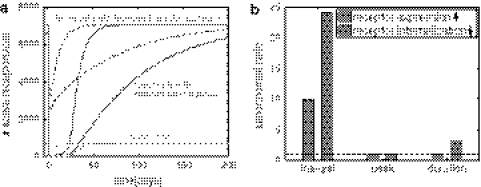
The mechanism underlying increased receptor levels influences tumor specificity of mAbs. a Predicted transient inhibition based on the extended cell-level pharmacokinetic model shown in Fig. 2d for normal cells, tumor cells with a 10-fold increased receptor expression, and tumor cells with a 10-fold decreased internalization of the free and bound receptor. Profiles are shown for the 20 mg/kg (*solid line*) and 2 mg/kg (*dashed line*) dose. Both scenarios show similar steady-state activation levels of the receptor, but their response to drug treatment is substantially different.** b** Antibody specificity as defined in Eq. 29. Cells with a decreased receptor internalization have a much longer duration of inhibition and therefore a higher integral of inhibition than tumor cells with an increased receptor expression

The cellular model for the tumor cells with increased receptor synthesis was chosen to resemble the characteristics of A431 cells, a human squamous carcinoma cell line with high EGFR levels [31– 33]. The overexpression in A431 cell is due to amplification of the EGFR gene [46] and correlates with increased EGF receptor mRNA levels [32]. A431 cells express about 10 times more EGFR at the cell surface than normal cells [31]. The cellular model for the tumor cells with decreased receptor internalization was chosen to resemble the characteristics reported in [34]. Reddy et al. [34] report about an alteration of EFGR where a truncated cytoplasmic domain exhibits a decreased ligand-induced internalization rate constant. Figure 5a illustrates the predicted inhibitory effect in tumor and normal cells in cynomolgus monkeys.

Figure 5b compares the predicted transient inhibition for both alterations, increased synthesis rate and reduced internalization. For both alterations, the inhibitory ef fect is strong er for tumor cells than for cells with nor mal EGFR levels. Although both cell alterations resulted in similar steady-state activation levels, their responses to mAbs are remarkably different with cells with decreased receptor internalization showing a higher integral and duration of inhibition compared to cells with an increased synthesis of the receptor.

## Discussion

The objective of this article was to develop a systematic approach to integrate the cellular-level into compartment models of drug PK, and to apply the approach to analyze the F(ab)-mediated inhibitory effect of therapeutic antibodies in cancer therapy.

Several mAbs on the market have a high receptor affinity in the sub-nM range, but the traditional design criterion that “the best binder makes the best drug” has been challenged [47–49]. Using our combined model we evaluated the effect of different affinities of antibodies targeting the EGFR. In cynomolgus monkeys, our cell-level PK/PD model predicts almost identical F(ab)-mediated direct inhibitory effects for a range of antigen-binding affinities. Since current anti-EGFR antibodies are located on the observed effect plateau, this relativizes the affinity amongst the properties that could be further tuned to optimize antibody efficacy.

A high affinity is thought to allow panitumumab to compete more effectively with EGF in binding to EGFR and to saturate EGFR in vivo at lower doses relative to mAbs with lower affinity [16]. This is not supported by our analysis, and instead our findings predict that the F(ab)-mediated effect of panitumumab and cetuximab are comparable. This prediction of the model is in agreement with experimental results by Messersmith and Hidalgo [50]. We further investigated if this result is due to the specific values of the parameters we used to simulate the model by calculating an analytically solution of the integral of the effect. The analytical solution shows that the existence of an effect plateau is a generic feature of this drug-target system and does not depend on specific parameter values. Therefore this result suggest that such an effect plateau might exist for other receptor systems with receptor trafficking.

Crombet et al. [48] argued that the low degree of adverse effects observed for Nimotuzumab in the clinics is due to its intermediate affinity compared to other anti-EGFR antibodies. Their conclusions are based on a mathematical model that only takes into account receptor binding, but neglects the important process of receptor internalization and target specific degradation. Based on our single-cell PK/PD model for cynomolgus monkeys, we find that an intermediate affinity does not result in optimized tumor effect or specificity. Recently, Talavera et al. [51] suggested an alternative explanation for the low degree of adverse effects observed for Nimotuzumab.

Based on the existence of an effect plateau in the F(ab)-mediated direct inhibitory effect, our findings suggest that the clinically observed differences among mAbs are likely to arise from Fc-mediated indirect effects, such as the action of immune effector functions (such as antibody dependent cell mediated cytotoxicity or complement dependent cytotoxicity), rather than the direct antagonistic effect. This is consistent with a study of Bleeker et al. showing that effects in vivo of zalutumumab and cetuximab differed only by their ability to trigger such indirect effect and not by their direct inhibitory effect [25]. Possible extensions of the model could address the likelihood of triggering such Fc-mediated indirect effects. Since the model predicts the time course of the different receptor species, it may serve as a starting point to estimate the proportion of bound antibody that are presented to the extracellular space and trigger Fc-mediated immune effects.

Alterations of a number of kinetic processes can result in elevated EGFR levels. The combined systems biology/ pharmacokinetic model allows us to study two different tumor cell alterations with elevated EGFR levels resulting from (i) an increased receptor synthesis rate; and (ii) a decreased receptor internalization rate. Both types have been observed experimentally [46, 32, 34]. We found that receptor inhibition over time strongly depends on the underlying molecular alteration that caused the elevated EGFR level.

Our in silico studies show that the inhibitory effects at normal and tumor cells are correlated, and therefore support the hypothesis that the side effects may serve as a marker for the desired effect at the tumor cells. This is in line with experimental observations that the most common side effect of anti-EGFR antibodies are cutaneous toxicities, affecting 45–100% of patients [52]. Since this skin rash follows from the inhibition of epidermal cells expressing normal levels of the EGFR, using the rash as a marker of drug activity and clinical outcome was proposed [53] and our theoretical study supports this.

The compartment model (1–3) describes the distribution of the drug to the target expressing cells. In this case we described distribution as reversible linear process as previously done when relating plasma concentration to potential pharmacodynamic effect [54]. However, processes such as convective movement, lymphatic circulation and filter effects can affect the distribution of antibodies. In these cases, detailed models for antibody distribution e.g. a two pore model (Rippe and Haraldsson, 1994, Physiological reviews) could be integrated into the model. Further, predictions of EGFR inhibition in tumor cells are limited to those malignant cells which are exposed to similar concentrations than normal cells, such as avascular metastases embedded in healthy tissue [55, 56]. In solid tumors, due to heterogeneous drug distribution, only malignant cells close to capillaries may be exposed to such concentration. Taken together, more detailed models of mAbs distribution, such as physiologically based pharmacokinetics models [57, 58], should be included in cases where distribution of the drug to target cells can not be described by reversible linear processes. The current model also predicts only the decrease in receptor activation rather than the actual biological response of the cell. While Knauer et al. [29] reported a linear dependence between the number of activated EGFR at steady-state and the cellular responses of fibroblasts and epithelial cells, other models describe a more complex relationship between receptor activation and downstream signalling [59].

Established models to study antibody pharmacokinetics include models of TMDD (e.g., Gibiansky et al. [8, 9]). Our cell-level PK/PD model has three important differences compared to TMDD models. First, the model includes the competition of natural ligands with the antibodies for the binding to the receptor. This allows us to study the change of the number of receptor-ligand complexes due to the drug treatment. Second, the model includes more details of the cellular mechanisms. For example, the internal pool of receptors and the recycling to the cell surface are part of the detailed receptor trafficking model, but not of current models of TMDD. We investigated if we could remove this pool together with receptor recycling to make the model more TMDD-like. However, we found this internal pool to be important to describe the initial PK without refitting of the experimentally derived parameters. Our findings support the hypothesis in [6] stating that “possibly, EGFR surface expression can temporarily be replenished with EGFR present in the cell”. Third, and most notably, our cell-level PK/PD model integrates in vitro determined parameter values instead of fitting all parameters to the in vivo data. This is useful to avoid over-paramerization of the model, which has be reported to be a critical problem when using the original TMDD model [8].

Combining modelling approaches from pharmacokinetics and systems biology allows us to quantitatively analyse the dynamic interaction between drugs and biological systems [15]. One remaining question concerns the validation of multi-level models. We here used the approach to validate the model prediction using pharmacokinetic data while integrating an in vitro validated cell-level model. Furthermore, we validated the full model using available PK data together with limited PD data. Ideally however, validation should be done using datasets that integrate pharmacokinetic and cell-level data (e.g. receptor phosphorylation) from one source.

We envision that a cell-level PK/PD modeling approach will prove valuable in the emerging field of systems pharmacology. The use of more detailed systems biology models describing downstream signaling processes relevant to human diseases [13, 60, 61] may eventually allow to translate plasma drug concentration into responses of tumor cells.

## Appendix

### Theoretical analysis of the integral of inhibition

The inhibitory effect can be studied by looking at the response of the model linearized around the steady state activation level. In what follows we derive a formula for the integral of inhibition in the linear system, which provides an estimate for that of the original system.

The steady state of model (20–40) is

$$ x^{*}= [C_{\rm int}^{*}\quad C_{\rm pla}^{*} \quad R_{\rm N}^{*} \quad R_{\rm i}^{*}\quad RL_{\rm N}^{*}\quad RC_{\rm N}^{*} \quad R_{\rm T}^{*} \quad R_{\rm i,T}^{*} \quad RL_{\rm T}^{*} \quad RC_{\rm T}^{*}], $$

and we know that *C*_int_^*^ = *C*_pla_^*^ = *RC*_N_^*^ = *RC*_T_^*^ = 0. We assume that the steady state is exponentially stable, which for any realistic scenario is trivially satisfied. This guarantees that the integral of the inhibition

41 $$ \begin{aligned}  E = \int\limits_{0}^{\infty} (RL_{\rm N}^{*} - RL_{\rm N}(t)) dt, \end{aligned} $$

is a finite number. The deviations of the model variables with respect to the steady state are

$$ \begin{array}{lll} \bar{R_{\rm N}} = R_{\rm N}^{*} - R_{\rm N} & \bar{R_{\rm T}}= R_{\rm T}^{*} - R_{\rm T} & \bar {C_{\rm pla}} = -C_{\rm pla}\\ \bar{R_{\rm i}} = R_{\rm i}^{*} - R_{\rm i} & \bar{R_{\rm i,T}} = R_{\rm i,T}^{*} - R_{\rm i,Y} & \bar {C_{\rm int}} = -C_{\rm int}\\ \bar{RL_{\rm N}}= RL_{\rm N}^{*} - RL_{\rm N} & \bar{RL_{\rm T}} = RL_{\rm T}^{*} - RL_{\rm T}\\ \bar{RC_{\rm N}}= - RC_{\rm N} & \bar{RC_{\rm T}} = -RC_{\rm T} \end{array} $$

We define a state vector as

42 $$ \begin{aligned}  \bar{x} = [\bar {C}_{\rm pla}\; \bar{C}_{\rm int}\; \bar{R_{\rm N}}\; \bar{R_{\rm i}}\; \bar{RL_{\rm N}} \;\bar{RC_{\rm N}} \;\bar{R_{\rm T}}\; \bar{R_{\rm i,T}}\; \bar{RL_{\rm T}} \;\bar{RC_{\rm T}}]^{T} \end{aligned} $$

Linearizing the model around the steady state leads to

43 $$ \frac{{\text{d}}\bar{x}}{{\text{d}}t} = {\user2{A}}\bar{x}. $$

The matrix ${{\user2{A}}\in\mathbb{R}^{10\times10}}$ is given in Eq. 53 and corresponds to the Jacobian of the right hand side of (30–40) evaluated at the steady state. Integration of (43) from *t* = 0 up to $t=\infty$ gives

44 $$ \begin{aligned} \bar{x}(\infty) - \bar{x}(0) = {\user2{A}} \int\limits_{0}^{\infty} \bar{x}(t)dt. \end{aligned} $$

For a bolus dose *C* at *t* = 0 the initial condition for (43) is

45 $$ \begin{aligned} \bar{x}(0) = [-C\;0 \; 0 \; \ldots\; 0 ]^{T}. \end{aligned} $$

The stability of the equilibrium implies that $\bar{x}(\infty)=0$, which upon substitution in (44) yields

46 $$ \begin{aligned} \int\limits_{0}^{\infty}\bar{x}(t)dt = -{\user2{A}}^{-1}\bar{x}(0).  \end{aligned} $$

From (42) we notice that the integral of inhibition *E* is the 5th entry of the vector in (46). Hence

47 $$ \begin{aligned}  E =\int\limits_{0}^{\infty}\bar{x}_{5}(t)dt= -[{\user2{A}}^{-1}\bar{x}(0)]_{5}. \end{aligned} $$

Computing the inverse ${\user2{A}}^{-1}$ we get

48 $$ E = {\frac{\alpha RL_{\rm N}^{*}}{\beta + \gamma {\frac{1}{k_{\rm onC}}}\left({\frac{1}{{\rm CP}}} + 1\right)}}, $$

with the constants:

49 $$ \alpha = V_{\rm pla}R_{\rm N}^{*} q_{\rm cp} C {\frac{k_{\rm degR}}{k_{\rm onL}L}}\left({\frac{k_{\rm offL} + k_{\rm degRL}}{k_{\rm recyRi} + k_{\rm degRi}}}\right), $$

50 $$ \begin{aligned} \beta = k_{\rm synR,N}\text {SF}_{\rm unit}(q_{\rm cp} + \text {CL}_{\rm lin})(N_{\rm N}R_{\rm N}^{*} + N_{\rm T}R_{\rm T}^{*})\end{aligned} $$

51 $$ \begin{aligned}\gamma = k_{\rm R,N}\text {CL}_{\rm lin}q_{\rm pc} \end{aligned} $$

The parameter *K*_d_ = 1/affinity and the “consumption parameter” defined by in [19] are given by

52 $$ \begin{aligned}K_{{\rm D}} = {\frac{k_{{\text{offC}}}}{k_{{\text{onC}}}}} \qquad{\text{CP}}\,=\,{\frac{k_{{\text{degRC}}}}{k_{{\text {offC}}}}}. \end{aligned} $$

The effect *E* is a decreasing function of *k*_offC_ and shows little variations for the values of *K*_D_ for the mAbs in Table 4. All these mAbs are located in a plateau region of the effect *E*. This linear analysis suggests that the effect plateau is a structural feature of the system and does not depend on the parameter values.

53 $$ \begin{aligned}{\user2{A}}=\left[\begin{array}{llllllllll} a_{11} &{\frac{q_{\rm pc}}{V_{c}}} & 0&0&0&0&0&0&0&0\\ {\frac{q_{\rm cp}}{V_{p}}}& a_{22} & 0&0&0&{\frac{k_{\rm offC}N_{\rm N}\text {SF}_{\rm unit}}{V_{p}}} &0&0&0& {\frac{k_{\rm offC}N_{\rm T}\text {SF}_{\rm unit}}{V_{p}}} \\ 0&-k_{\rm onC}R_{\rm N}^{*}&-k_{\rm onL} L-k_{\rm degR}&k_{\rm recyRi}&k_{\rm offL}&k_{\rm offC}&0&0&0&0\\ 0&0&k_{\rm degR}&-k_{\rm recyRi}-k_{\rm degRi}&0&0&0&0&0&0\\ 0&0&k_{\rm onL}L&0&-k_{\rm offL}-k_{\rm degRL}&0&0&0&0&0 \\ 0&k_{\rm onC}R_{\rm N}^{*}&0&0&0&-k_{\rm offC}-k_{\rm degRC}&0&0&0&0\\ 0&-k_{\rm onC}R_{\rm T}^{*}&0&0&0&0&-k_{\rm onL}L-k_{\rm degR}&k_{\rm recyRi}&k_{\rm offL}&k_{\rm offC}\\ 0&0&0&0&0&0&k_{\rm degR}&-k_{\rm recyRi}-k_{\rm degRi}&0&0\\ 0&0&0&0&0&0&k_{\rm onL}L&0&-k_{\rm offL}-k_{\rm degRL}&0\\ 0&k_{\rm onC}R_{\rm T}^{*}&0&0&0&0&0&0&0&-k_{\rm offC}-k_{\rm degRC}\\ \end{array}\right]\end{aligned}$$

54 $$ a_{11} = {\frac{-1}{V_{c}}} ( q_{\rm cp}+CL_{\rm linC}) $$

55 $$ a_{22} = {\frac{-1}{V_{p}}}( k_{{\rm onC}}N_{{\rm N}}{\text{SF}}_{{\text{unit}}}R_{{\rm N}}^{*} + k_{{\rm onC}}N_{{\rm T}}{\text{SF}}_{{\text{unit}}}R_{{\rm T}}^{*} + q_{{\rm pc}}) $$

## Footnotes

1 We transformed the originally published system of difference equations [6, Supplement] into a corresponding continuous system of ordinary differential equations. The originally published equations in [6, Supplement] are identical to a certain discretization of the system of ODEs (1–3). The advantage of stating the system as continuous ODEs is that subsequently any numerical scheme can be used to solve them, in particular high accuracy ODE solver with adaptive step size control. See also [28].
